# Book Reviews, Pamphlets, Exchanges

**Published:** 1901-08

**Authors:** 


ECZEMA, WITH AN ANALYSIS OF 8,000 CASES OF THE DISEASE. By L. Duncan Bulkley, M.D., Physician to the New York Skin and Cancer Hospital; Dermatologist to the Randall's Island Hospitals; Consulting Physician to the New York Hospital, Hospital for Ruptured and Crippled, and Manhattan Eye and Ear Hospital, etc. Third edition of Eczema and its Management. Entirely rewritten. G. P. Putnam's Sons, New York and London, 1901.

This is a very practical and useful book and destined to accomplish much good. Eczema is a disease but little understood by the general practitioner and the term is used to cover many and manifold cutaneous ills. A perusal of this volume will be in the nature of an illumination to him and will enable him to cope intelligently with an affection which cuts no small figure in the bulk of his practice. To the dermatologist it is quite acceptable as the views expressed are orthodox and recent.

FAVORITE PRESCRIPTIONS OF DISTINGUISHED PRACTITIONERS, WITH NOTES ON TREATMENT. Edited by Dr. E. W. Palmer. Seventh edition. New York, $2.00. E. B. Treat & Co.

This small volume will be found of avail as a reference book for combinations of approved efficacy. The prescriptions are all classified and are easily gotten at. The young practitioner can get much valuable information from studying the drug combinations with which the sick have been dosed with varying success for the last fifty years.

---

ANNUAL AND ANALYTICAL CYCLOPEDIA OF PRACTICAL MEDICINE. Vol. VI. The F. A. Davis Co., Phila.

Sajous' annual is already so widely and favorably known that it scarcely needs further commendation here. The present volume is the final one and completes the alphabetical list of diseases. It is an invaluable addition to a doctor's library and is a monument to the progress of medicine.

---

### DIFFERENTIAL DIAGNOSIS IN DISEASES OF THE BREAST.

If a lump is found in the breast, the question arises, says J. O. Warren (*Annals of Gynecology and Pediatry*, May, 1901), of a differental diagnosis between cancer and other disease. We must, says Warren, keep steadfastly in mind the period of life in which the patient is, and the probability of other diseases of that period being present in this case. The ill-defined character of the lump is suggestive of cancer, and also, of more importance still, the presence of glands in the axilla. In hardly any other disease are enlarged and indurated glands to be found in the axilla. Small glands may be found that are perceptible to the touch in almost every axilla, some of them perhaps tolerably hard, but if distinct glands matted together with induration are found, it is a suggestive symptom. The presence then of a lump in the upper outer quadrant and glands in the axilla of a woman forty-five years of age will be almost pathognomonic of the disease. And yet it is possible that the nodules may be a cyst, because cysts are so hard that they often pass for an indurated mass. Although the substance of the cyst may be hard, its outline is much better defined than that of a cancer, and cancer must not be regarded as a tumor of the breast, but an infiltration of the breast tissue. When the disease has progressed further, the affection is much more easily recognized.—*Medical Age.*

